# Model of Selective and Non-Selective Management of Badgers *(Meles meles)* to Control Bovine Tuberculosis in Badgers and Cattle

**DOI:** 10.1371/journal.pone.0167206

**Published:** 2016-11-28

**Authors:** Graham C. Smith, Richard J. Delahay, Robbie A. McDonald, Richard Budgey

**Affiliations:** 1 National Wildlife Management Centre, Animal and Plant Health Agency, Sand Hutton, York, United Kingdom; 2 Environment and Sustainability Institute, University of Exeter, Penryn Campus, Penryn, Cornwall, United Kingdom; University of Minnesota, UNITED STATES

## Abstract

Bovine tuberculosis (bTB) causes substantial economic losses to cattle farmers and taxpayers in the British Isles. Disease management in cattle is complicated by the role of the European badger (*Meles meles*) as a host of the infection. Proactive, non-selective culling of badgers can reduce the incidence of disease in cattle but may also have negative effects in the area surrounding culls that have been associated with social perturbation of badger populations. The selective removal of infected badgers would, in principle, reduce the number culled, but the effects of selective culling on social perturbation and disease outcomes are unclear. We used an established model to simulate non-selective badger culling, non-selective badger vaccination and a selective trap and vaccinate or remove (TVR) approach to badger management in two distinct areas: South West England and Northern Ireland. TVR was simulated with and without social perturbation in effect. The lower badger density in Northern Ireland caused no qualitative change in the effect of management strategies on badgers, although the absolute number of infected badgers was lower in all cases. However, probably due to differing herd density in Northern Ireland, the simulated badger management strategies caused greater variation in subsequent cattle bTB incidence. Selective culling in the model reduced the number of badgers killed by about 83% but this only led to an overall benefit for cattle TB incidence if there was no social perturbation of badgers. We conclude that the likely benefit of selective culling will be dependent on the social responses of badgers to intervention but that other population factors including badger and cattle density had little effect on the relative benefits of selective culling compared to other methods, and that this may also be the case for disease management in other wild host populations.

## Introduction

Wild animals act as the reservoir of many infectious diseases that can be transmitted to domestic livestock or humans [1]. The most common approach to managing disease in wildlife is culling [2] and for a limited number of diseases vaccination of the wild host is also possible [3]. In some scenarios culling may not be effective for disease management (e.g. culling to control infectious cancer in Tasmanian devils [4]) and may even increase disease persistence (e.g. longer term persistence of rabies in the presence of fox culling [5]).

Bovine tuberculosis, bTB, caused by *Mycobacterium bovis*, is a serious economic disease of cattle and in the British Isles its management in livestock is complicated by the involvement of the European badger (*Meles meles*), which may be responsible for about half of all cases in cattle [6]. Widespread and sustained badger culling can reduce bTB incidence in cattle herds in culled areas in England [7] and in the Republic of Ireland [8]. Localised or reactive badger culling in response to infection on individual farms has, however, been associated with no benefit, or some level of detrimental effect on bTB incidence in cattle herds in both England [9] and Ireland [10]. In England, widespread badger culling has also been associated with an increased incidence of bTB in cattle herds in the area surrounding the culling area [11], although this negative outcome has not been reported in Ireland [8]. The detrimental increase in herd incidence observed in England is believed to arise because of culling-induced perturbation of the otherwise stable social structure in badger populations, which is characterised by increased ranging behaviour and thus more opportunities for disease transmission [12]. The reason that this effect was not recorded in Ireland could potentially relate to geographic differences such as the configuration of cattle farms and badger social group size [13].

Vaccination of badgers with Bacillus Calmette-Guérin (BCG) has been demonstrated to reduce the severity and progression of bTB infection in badgers [14], and to provide indirect protection of badger cubs [15]. Vaccination may therefore have a role to play in disease control in badgers although it has not yet been empirically tested what level of effect this treatment of badgers would have on bTB incidence in cattle. Given the contentious nature of badger culling as a means of controlling bTB in cattle, combined application of selective culling of infected badgers and vaccination of the remainder is potentially a useful and more socially acceptable approach that is worthy of investigation. The disease control benefits of such an approach will depend in part on the levels of social perturbation that arise when animals are removed [11], how many badgers are trapped and tested, and the performance of the test to correctly identify infected animals [13, 16]. Recent analysis suggests that even very low levels of culling may be sufficient to produce measurable social perturbation in badgers [17]. Here, we use an established simulation model [18] to investigate the expected effect of a selective test and vaccinate or remove (TVR) approach on infection in badgers and cattle. We also investigate the effects of variation in host (badger and cattle) density.

## Methods

### Model Description

We used an established simulation model to predict the efficacy of three distinct interventions in badgers with the objective of controlling levels of bTB in both badger and cattle populations: non-selective badger culling, non-selective vaccination and the selective TVR approach. The model and the rationale behind its design and development are outlined here and described in detail elsewhere [19–23]. A sensitivity analysis which determined the effect of varying input parameter values on the resultant badger prevalence and herd breakdown rate, and once an appropriate range of values was identified, on the efficacy of the chosen control strategy has also been undertaken previously [18], with a full description of the model and parameter values in S1–S4 Appendixes.

### Overview

We used a quasi-individual based model that simulated the population dynamics of wild badgers and cattle farming practices, and the dynamics of bTB within and between each host population. The efficacy of a range of badger control methods to reduce bTB in cattle was investigated. The model’s starting conditions were set to represent high bTB incidence regions within the United Kingdom (UK): South West England and Northern Ireland. The two regions and their associated disease dynamics were simulated for a number of years to stabilise before any intervention was implemented. By default, interventions occurred for five years across approximately 100 km^2^ within a total model area of 400 km^2^. Results were recorded for a range of measures including the numbers of badgers removed, the incidence of bTB in cattle herds and the prevalence of bTB in badgers.

By comparison with an earlier published version of the model [18], badger mortality rates and disease state definitions were updated on the basis of recent work [24]. Simulations explored the impact of social perturbation of badger populations extending over different spatial scales and temporal duration until the model outcomes best matched the latest analysis of cattle herd breakdown (CHB) rates over time from the Randomised Badger Culling Trial (RBCT), an extensive field-based investigation into the effects of badger culling in South West England [7]. The closest approximations to the RBCT outcomes were observed when the model assumed that perturbation in badgers lasted for 12 months after culling started, extended its influence over two badger group territories away from culled groups, resulted in increased between-group transmission amongst badgers to the same level as that seen within social groups, and permitted badgers to infect cattle in neighbouring herds at 30% of the rate of infection to herds within their territory. Although this period of enhanced transmission was simulated for only 12 months, the effects of perturbation in terms of increased prevalence of bTB in badgers was found to last a total of four years. We assumed badger behaviour was identical in both locations with respect to social perturbation.

### Organisation of the model

The model was spatially explicit with badger territories, farms and administrative areas (parishes) organised by combining cells on a grid representing the landscape, and all population dynamics, disease and population control processes were spatially organised within territories, farms or parishes. The grid was wrapped round to form a torus to eliminate edge effects. The spatial area components were distributed randomly at a realistic density and shape, and new spatial configurations and initial host populations were created for each iteration. Processes such as badger dispersal, culling-related perturbation of badgers and cattle movements (trading) also occurred. Cattle were either dairy or beef and farms may include either or both types. Farms had an appropriately sized contiguous area of randomly positioned grazing, permitting some direct disease transmission between neighbouring herds, and also accounting for non-grazing land within farms. The simulation was divided into three areas: the intervention area or core area where badger management occurred; the outer ring immediately surrounding the core with a width equivalent to two badger social groups, akin to the 2km peripheral zones analysed in the RBCT [11], and the remaining, non-treatment area.

All events occurred within defined time steps of two months. Most were controlled by parameters that were randomly varied within permitted ranges, allowing stochasticity within the model. The model was not fully individual-based, in that it did not keep track of the fate of individuals, but the number of individuals of each sex, age, infection and vaccination status within each badger social group or cattle herd was monitored.

### Population dynamics

The badger population was regulated by parameters including mortality and birth rate, several of which (principally social group density and group carrying capacity) were manipulated to produce badger populations with characteristics matching those in the region of interest [22]. Equivalent parameters for the cattle population included herd sex and age composition, stocking density, birth and mortality rates and the likelihood of cattle being traded between farms. The herd characteristics were adjusted to match those found in the region under study [22]. The model simulated cattle movements such that approximately 40% of animals moved each year [18]. If at any point a herd had lower or higher stocking density than the starting point, then extra movements occurred to redress the balance. Cattle that were slaughtered, either routinely or because they tested positive for infection, were removed from the population. Natural mortality was not considered as it was negligible compared to slaughter.

The three intervention approaches and no badger control were modelled under two scenarios each of which represented cattle farming and badger density characteristics typically found in South West England and in Northern Ireland.

### Disease dynamics

The model permitted disease transmission between individuals within host populations and in both directions between populations, dependent on infection status [18]. Within the badger population, there were different disease transmission rates within and amongst social groups to represent respective contact rates, and a very much lower rate for transmission between badgers and cattle. Manipulation of the (unknown) transmission rates resulted in realistic spatial and temporal disease prevalence in both badgers and cattle. In England, prevalence in the badger population stabilised at 18% and the CHB rate at 6.5% per farm per year [22], and in Northern Ireland, the rates supplied to us by the Department of Agriculture, Environment and Rural Affairs (DAERA) were 15% and 7.3% respectively.

The progression of disease within each population and the response to infection within a herd (movement restrictions, more stringent testing regime) were also modelled, allowing the maintenance and spread of the disease to be followed and the effect of any badger intervention to be investigated.

Cattle were routinely tested for bTB at an interval determined by the local (parish level) CHB rate using the decision process used by Defra prior to 2013, although this policy has subsequently been changed to one of requiring annual routine testing for all farms in this region. The detection of bTB infection in cattle was determined probabilistically to simulate the sensitivity and specificity of the skin test, and positive tests triggered slaughter, *post mortem* examinations, movement restrictions and follow-up testing of any contacts. Pre-movement testing was simulated in South West England but not Northern Ireland. This required that in areas already requiring one-or two-yearly routine bTB tests, cattle over a certain age had to have a recent negative bTB test before being allowed to move from one farm to another. All cattle were tested at routine slaughter, although the detection probability of infected animals was low (0.217) and if TB was detected, movement restriction and testing was triggered at the farm of origin.

### Badger management

The model simulated both cattle and badger populations for a number of years until they stabilised. All data were then stored so that a number of different management options could be run from an identical starting point. Modelled disease management interventions in badgers targeted those parishes with the most CHBs during the previous three years. Farms around the selected location participated in the badger interventions at a given probability of compliance until 100 km^2^ was reached. The proportion of compliant accessible land within the intervention area was set to 70% to maintain consistency with the RBCT [25] and subsequently licensed culls [26, 27]. Annual trapping efficacy of badgers was set at 70% to simulate field estimates for this method [28]. Badger social groups that were situated within or partly within participating farms in the intervention area were identified and subjected to intervention.

Non-selective badger culling, non-selective vaccination and TVR were all simulated once per year for a five year period. In the non-selective culling scenario, animals were removed from the population at random at a probability determined by trapping efficacy. For the non-selective vaccination scenario, animals were trapped with the same probability, then vaccinated and released. Each time a healthy badger was vaccinated it was given a 70% probability of becoming fully and permanently protected against bTB [14, 15]. It was assumed that vaccination had no effect on an animal that was already infected with bTB. For TVR, animals were trapped with the same probability as above and tested with a test, the performance of which was equivalent to the badger bTB anti-body test, the Brock-TB StatPak which was assumed to be able to provide a test result in the field and for which sensitivity and specificity values obtained in laboratory studies [29]. Those badgers that tested negative were vaccinated and released while those that tested positive were culled.

### Social Perturbation

It is not currently known whether there is a threshold, in terms of a proportion or number of social group members removed, below which social perturbation does not occur [17]. If such a threshold exists then perturbation would not be expected to occur as a result of TVR where sufficiently small numbers of animals were removed. The model was therefore run with perturbation switched on for all management approaches, and then re-run for TVR without perturbation, so as to represent minimum and maximum impacts of perturbation for TVR, with the likelihood that in reality we would expect results to lie between the two.

### Data output

The model was run with 100 simulations from the same starting conditions. Results from each intervention, and a scenario where no action on badgers was taken were recorded separately for the whole grid (to account for social perturbation) and for the intervention areas. Key output parameters were mean badger social group size, mean number of infected badgers per social group and cattle herd breakdown rate per farm. While the cattle herd incidence rate is the ultimate measure of success for any disease management in badger populations, it may be a less informative measure of the relative effects of badger management scenarios than changes in the absolute number of infected badgers per social group, owing to the additional model stochasticity in modelling cattle infections. In addition, we expect any simulation to be less accurate as it projects further into the future, so we present our results separately for the five years of intervention and the subsequent five years.

## Results

The benefits achieved by the four active management strategies (non-selective culling, non-selective vaccination, TVR with perturbation and TVR without perturbation) when compared to taking no action (no control) are presented here, as is the relative benefit of each method, under different area, control phase and region. Results are presented for badger infection levels and CHB rates. In most cases, there was a benefit from all methods when compared to no control. The benefit of TVR on both badger infection levels and CHB rates was highly dependent on whether perturbation was invoked as this generally caused it to switch from being the best to the worst option. The effect of any strategy on badger infection levels was always more substantial than on CHB rates. There was little difference between all strategies in their effect on badger or cattle infection rates between England and Northern Ireland.

### Comparison between regions

The different input data for South West England and Northern Ireland resulted in a substantial difference in herd density and badger social group density (Table 1). The cattle herd density in Northern Ireland was twice that in England, while the badger density (product of social group size and number of social groups) in England was twice that in Northern Ireland. The rate of confirmed CHBs under the no-control scenario was slightly higher in Northern Ireland than in England.

**Table 1:** The different numbers of farms and badger social groups in the simulated Northern Ireland (NI) and England areas (used as input), and the corresponding starting levels of infection in badgers and cattle (from simulation results).

	NI	England
**No. farms**	701	312
**No. badger social groups**	224	300
**Mean badger social group size**	4.4	6.7
**TB prevalence rate in badgers**	15%	18%
**Mean infected badgers per group**	0.69	1.26
**Annual cattle herd incidence rate (CHB)**	0.073	0.065

There was however, little difference between England and Northern Ireland within the intervention area on the relative effects of the different strategies on either badger infection levels or CHB rates. When the whole simulation area was considered, the effect of control methods that triggered perturbation (non-selective culling and TVR with perturbation) produced little benefit on CHB rates during control but some benefit afterwards in England, but in Northern Ireland they were associated with increased CHB rates during control and no benefit afterwards.

### Comparison between areas

Within the 100km^2^ intervention area, the non-selective badger culling scenario removed a mean of 488 badgers in NI and 982 in England during the five years of intervention. TVR removed a mean of 82 in NI and 160 in England when perturbation was enacted. In both cases the TVR approach resulted in an 83% reduction in the number of badgers killed, compared to non-selective culling. Non-selective culling resulted in a greater reduction in the number of infected badgers and the CHB rate than non-selective vaccination both during and after intervention, regardless of location. The benefits of TVR for cattle herd incidence of bTB were generally greater than non-selective culling if there was no perturbation, but worse than non-selective vaccination in the presence of perturbation.

In the 2 km ring around the intervention area, non-selective vaccination had a very small benefit in terms of reducing the number of infected badgers, presumably due to a reduced force of infection and the occasional migration of vaccinated badgers into this area. There was negligible effect on the CHB rate. In this area, non-selective culling resulted in an increase in the number of infected badgers and the CHB rate during the intervention, but a subsequent benefit once it ceased. TVR had a negligible benefit on infected badgers and CHBs without perturbation but with perturbation was worse than non-selective culling.

Negligible effects on the output metrics were seen for non-selective culling, non-selective vaccination and TVR at distances greater than 2 km from the intervention area.

### Comparison between management strategies

**Fig 1:**
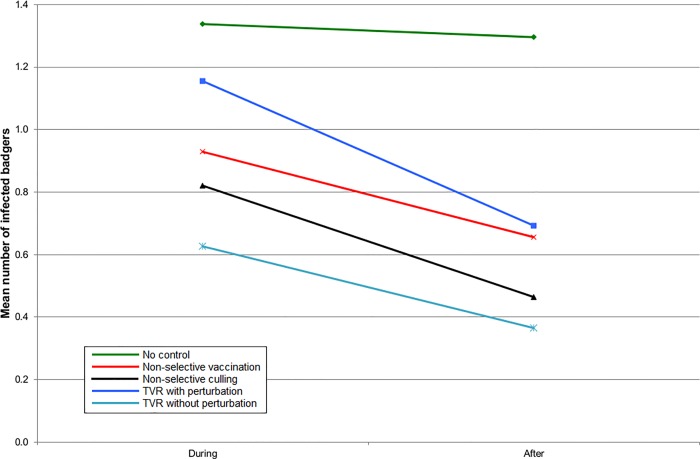
The mean number of infected badgers per social group during the five years of control and five years afterwards inside the control area for simulations representing SW England.

**Fig 2:**
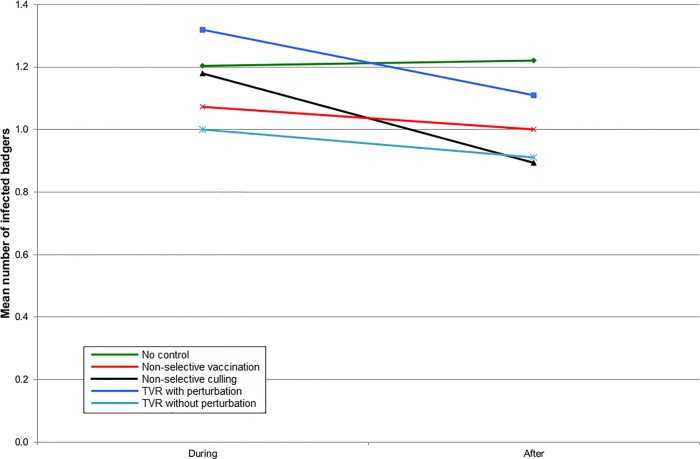
The mean number of infected badgers per social group during the five years of control and five years afterwards within the entire simulation area for simulations representing SW England.

**Fig 3:**
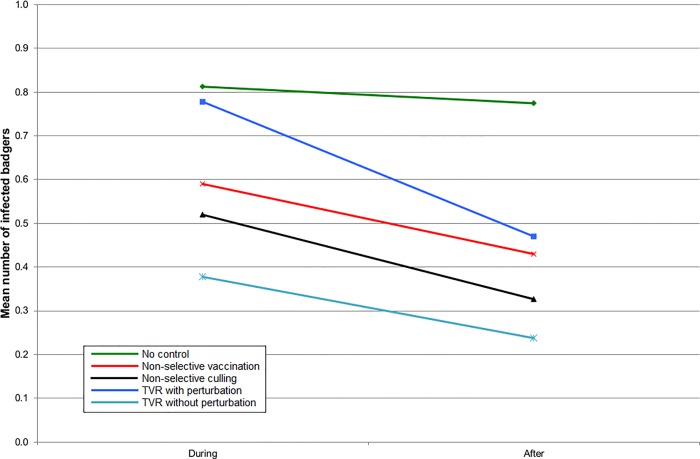
The mean number of infected badgers per social group during the five years of control and five years afterwards inside the control area for simulations representing Northern Ireland.

**Fig 4:**
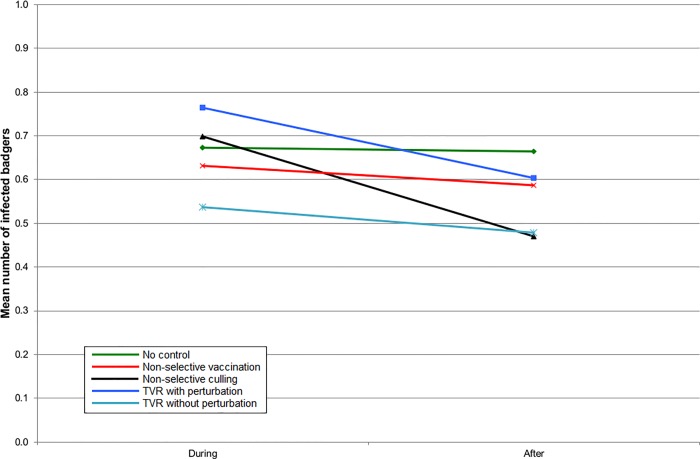
The mean number of infected badgers per social group during the five years of control and five years afterwards within the entire simulation area for simulations representing Northern Ireland.

In England, all the intervention strategies reduced the number of infected badgers inside the intervention area (Fig 1), but when the surrounding area was also included, the benefits of non-selective culling became approximately comparable to non-selective vaccination (Fig 2). TVR with perturbation gave no benefit at all when averaged over the ten year period (Fig 2). TVR without perturbation gave an outcome slightly better than non-selective culling (Fig 2). The results were almost identical for Northern Ireland, although the number of infected badgers was less in all cases (Figs 3 and 4).

**Fig 5:**
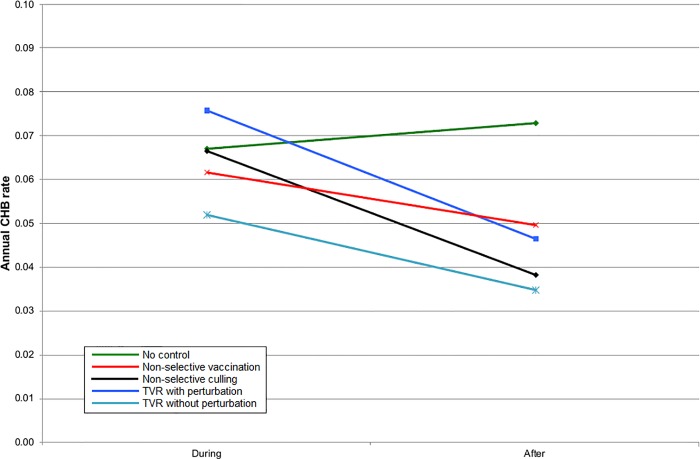
The mean cattle herd breakdown (CHB) rate during the five years of control and five years afterwards inside the control area for simulations representing SW England.

**Fig 6:**
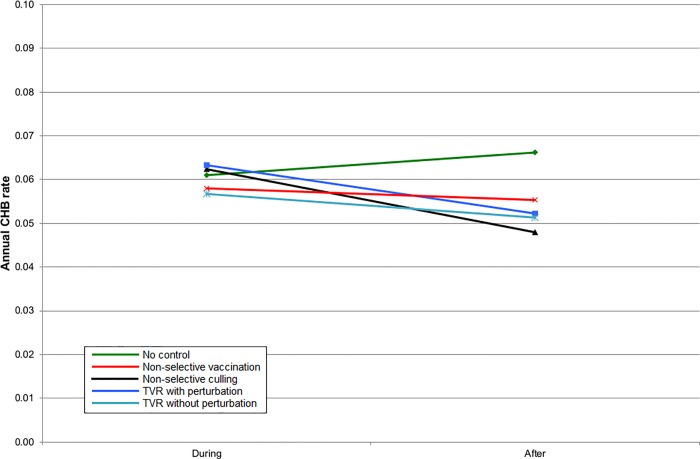
The mean cattle herd breakdown (CHB) rate during the five years of control and five years afterwards within the entire simulation area for simulations representing SW England.

**Fig 7:**
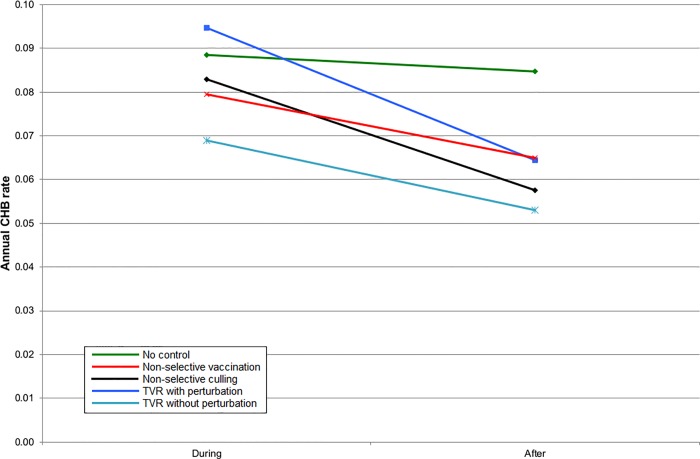
The mean cattle herd breakdown (CHB) rate during the five years of control and five years afterwards inside the control area for simulations representing Northern Ireland.

**Fig 8:**
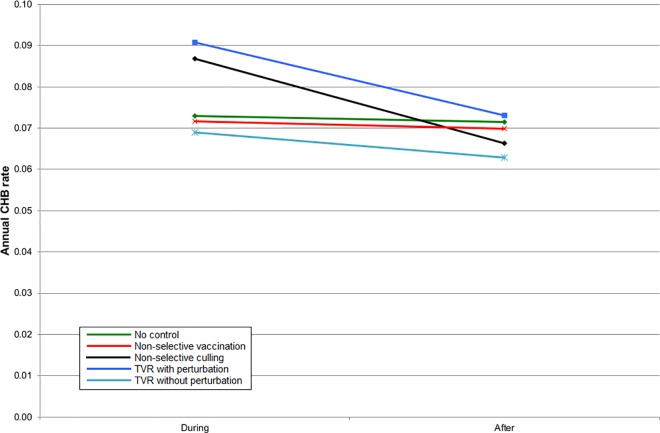
The mean cattle herd breakdown (CHB) rate during the five years of control and five years afterwards within the entire simulation area for simulations representing Northern Ireland.

Given that badgers cause only a proportion of CHBs, the benefit of all badger intervention strategies on the CHB rate was proportionately reduced. In England, all strategies gave a benefit within the intervention area, although for TVR with perturbation, this was only apparent once culling had ceased (Fig 5). When the intervention area and surrounding areas were considered together then non-selective culling and TVR with perturbation gave a small disadvantage during implementation but non-selective culling provided the greatest subsequent benefit. However, there was very little to choose between any of the strategies in terms of impacts on CHBs (Fig 6). The results for Northern Ireland were very similar to England within the intervention area (Fig 7). Overall in Northern Ireland, there was greater variation in the CHB rate between strategies, with both non-selective culling and TVR with perturbation leading to disadvantages when averaged over ten years, and non-selective vaccination and TVR without perturbation leading to small advantages (Fig 8).

## Discussion

The selective removal of individual infected animals is a potential tool for the management of disease in wildlife populations [2, 30]. The approach may have some public support as a tool to control bTB in badgers and cattle [31]. Although an intuitively appealing approach, a selective culling trial was not able to reduce the prevalence of Devil Facial Tumour Disease in the Tasmanian devil [4]. Furthermore, retrospective analyses of badger culling data suggest that even the removal of small numbers of animals may be associated with measurable perturbation of some elements of badger social behaviour, thus raising questions over the likely benefits of selective culling [17]. In principle, vaccination might offset some of the potential downsides of culling, though more selective approaches are constrained by practical limits on trap success, test performance and vaccine efficacy. Selective culling and vaccination of badgers was considered but not supported as a policy option in Wales [32], whereas in Northern Ireland, a selective Test-Vaccinate-Release approach is currently under field investigation [33].

In the present study we used an established model to simulate bTB dynamics in badger and cattle populations seen in two distinct areas: South West England and Northern Ireland. The cattle herd breakdown rate was slightly higher in Northern Ireland, whereas the simulated number of infected badgers per social group was lower. A number of badger management interventions were then simulated: selective culling and vaccination with and without social perturbation of badgers, non-selective culling and vaccination. Assuming similar levels of capture efficiency (i.e. 70%), the selective culling and vaccination strategy resulted in substantial reduction in the number of badgers culled (83% less) when compared to non-selective culling. Such a reduction, which almost exclusively removes infected individuals, since the diagnostic tests are highly specific even though sensitivity is much lower [29] may be more publicly and ethically acceptable, but will incur additional economic costs of testing and vaccination.

Culling-induced social perturbation of badgers in the model was parameterised [18] such that the associated increase in CHB rates matched findings from the Randomised Badger Culling Trial [7]. We found that this also resulted in a short-term increase in CHB rate that switched to a benefit immediately after culling ceased similar to that seen in the RBCT [7]. We can therefore be fairly confident that the overall results for non-selective culling are reasonably accurate for England and by extension for Northern Ireland.

Although there is evidence of a positive effect of vaccination on TB progression in badgers [15] there are no empirical data on how vaccination of badgers impacts on cattle herd incidence. However, badger vaccination initiatives have now been underway for several years, with the number of doses deployed exceeding 4000 in Wales [34] and over 5800 in England (APHA, unpublished data) by the end of 2014. Over time these projects may yield data that could be used to determine whether there has been a response in CHB rates, and to improve the estimates of vaccine efficacy in the model.

A selective or low-level badger culling strategy has not yet been tested in the field to permit the measurement of any resultant social perturbation. Limited evidence to date has raised the possibility that even low level culling, as would occur with a TVR approach, could potentially lead to some level of measurable social perturbation [17] although the consequences for CHB rates are unknown. Interpolating from the two extremes for TVR with and without perturbation, the simulations presented here indicate that TVR with limited perturbation could be similar to non-selective culling or non-selective vaccination in terms of the number of infected badgers remaining per social group. The lower number of animals removed by TVR should make this strategy more socially appealing. However, it also means that more animals are left alive and perturbation of their social behaviour could potentially compromise control. If, however, the actual level of perturbation is less than occurred during the RBCT, or is in an aspect of behaviour that has little bearing on the spread of infection, then the number of infected badgers remaining may be expected to lie somewhere between our two simulated scenarios and a benefit similar to non-selective culling or non-selective vaccination may occur, but with a much reduced number of animals killed.

The density of badger social groups is lower and the density of cattle herds is higher in Northern Ireland than in South West England and this may account for the larger difference among badger management strategies in terms of outcomes for cattle (compare Figs 6 and 8). However, it is encouraging that the general results appear to be the same regardless of these differences. Thus TVR may be a better strategy where perturbation is, or can be, reduced. The variation in individual iterations was quite large [13] which further suggests that a real difference may be more likely to occur in Northern Ireland.

The relative area of badger perturbation could be reduced for any culling strategy by increasing the area of control and thus reducing the relative size of the outer ring of perturbed badgers and by carefully choosing intervention areas that have hard boundaries, such as major rivers and coastline. Such an approach could further improve selective and non-selective culling strategies relative to vaccination.

Despite some public support for an intuitively appealing selective culling strategy there is no clear evidence from this modelling exercise that this approach would produce better results for cattle infections than non-selective culling or non-selective vaccination. Thus any benefits of a selective approach stem from reductions in the numbers of badgers killed, which must be weighed against potential increases in the costs of implementation. There remains a risk that selective approaches could make outcomes for cattle worse, should social perturbation lead to increased transmission of infection among badgers in a badger population that is not greatly reduced in density. The role of social perturbation is likely to be pivotal in influencing epidemiological outcomes. Hence, field evidence for the levels of culling-induced social perturbation and epidemiological consequences of TVR in badger populations would provide valuable data to more confidently predict outcomes by simulation modelling. Our findings are likely to have wider relevance to the control of infectious disease in other species, as they suggest potentially disparate outcomes to management interventions depending on host population responses. Recent empirical findings in deer indicate that resultant behaviour may also vary by age or sex [35].

## Supporting Information

S1 Appendix Model Variables (Temporal Settings). (DOC)

S2 Appendix Model Variables (Spatial Settings). (DOC)

S3 Appendix Model Variables (Badger and Cattle Parameters). (DOC)

S4 Appendix Model Processes (submodels). (DOC)
