# Supplementary material for: Model of Selective and Non-Selective Management of Badgers (Meles meles) to Control Bovine Tuberculosis in Badgers and Cattle
Source: PLoS One. 2016 Nov 28;11(11):e0167206. doi: 10.1371/journal.pone.0167206 (PMC5125688; doi:10.1371/journal.pone.0167206)
Supplement: S1 Appendix — (DOC) [file pone.0167206.s001.doc]

# S1 Appendix – Model Variables (Temporal Settings)

| Year Badgers added to grid | 1 |
| --- | --- |
| Year cattle added to grid | 20 |
| First Year that routine Test Interval switching is introduced (where parishes can change status from one test-interval type to another if CHB rate low or high).  The algorithm to determine switching is as follows:  1. Take the confirmed breaks in a parish over the previous 2 years and divide by the total herds in the parish, if the % is over 1 then the testing interval is yearly (T1)   1. If not (1) then do as above but consider breaks over 4 years, if the % is greater than 0.2 then the interval is 2 yearly (T2) 2. If not (2) then do as above but consider breaks over 6 years, if the % is greater than 0.1 then the interval is 3 yearly (T3) 3. If none of the above then the interval is 4 yearly (T4)   This algorithm was supplied by the AHVLA, and is the one advised by the EU, and used in the field. | 50 |
| First year that Pre-Movement Testing (PrMT) is introduced | 100 |
| Years of Badger Control | 120-124 |
| Years that badger perturbation is applied (cull options only) | 120-127 |
| Last Year of each simulation | 160 |
