# Supplementary material for: Model of Selective and Non-Selective Management of Badgers (Meles meles) to Control Bovine Tuberculosis in Badgers and Cattle
Source: PLoS One. 2016 Nov 28;11(11):e0167206. doi: 10.1371/journal.pone.0167206 (PMC5125688; doi:10.1371/journal.pone.0167206)
Supplement: S2 Appendix — (DOC) [file pone.0167206.s002.doc]

# S2 Appendix – Model Variables (Spatial Settings)

|  | **England** | **NI** |
| --- | --- | --- |
| Target Control Area (km^2^) | 100 | 100 |
| Grid size (squares per side) | 100 | 100 |
| Grid-Cell size (km) | 0.2 | 0.2 |
| Grid Area (km^2^) | 400 | 400 |
| Total Parishes | 30 | 30 |
| Parishes subject to Control | 7 | 7 |
| Mean Parish Size (km^2^) | 13.3 | 13.3 |
| Mean Control Area (km^2^) | 100 | 100 |
| Control Area as proportion of simulation grid | 0.25 | 0.25 |
| Badger Groups | 300 | 224 |
| Mean Badger Territory Size (km^2^) | 1.33 | 1.78 |
| Beef farms | 70 | 375 |
| Dairy farms | 58 | 82 |
| Mixed farms | 18 | 21 |
| X4 farms - mixed other species (mainly cattle) | 37 | 185 |
| X3 farms | 37 | 3 |
| X2 farms | 37 | 23 |
| X1 farms - mixed other species (mainly others) | 37 | 2 |
| Total Farms | 312 | 701 |
| **Grazing proportions** | **England** | **NI** |
| Beef farms | 0.26 | 0.54 |
| Dairy farms | 0.46 | 0.95 |
| X4 farms | 0.20 | 0.69 |
| X3 farms | 0.15 | 0.95 |
| X2 farms | 0.10 | 0.67 |
| X1 farms | 0.05 | 0.82 |

Farm numbers and grazing proportions calculated from June Census 2004. Farms mixed with other species (pigs, sheep, etc.) divided equally between X1-X4. Mixed farms have both dairy and beef comprising two smaller grazing areas. X1-X4 farms represent farms that also have other stock and hence are allocated as beef or dairy, but with proportionately smaller grazing areas for cattle.
