# Supplementary material for: Model of Selective and Non-Selective Management of Badgers (Meles meles) to Control Bovine Tuberculosis in Badgers and Cattle
Source: PLoS One. 2016 Nov 28;11(11):e0167206. doi: 10.1371/journal.pone.0167206 (PMC5125688; doi:10.1371/journal.pone.0167206)
Supplement: S3 Appendix — (DOC) [file pone.0167206.s003.doc]

# S3 Appendix – Model Variables (Badger and Cattle Parameters)

**Appendix 3.1** Badger Settings:

| **Initial Badgers Added per social group**  Figures obtained by iterative process to give ratio of badger ages and sexes that the model stabilizes at. | **England** | **NI** |
| --- | --- | --- |
| Juvenile male | 0 or 1 (mean 0.8) | 0 or 1 (mean 0.4) |
| Yearling male | 0 or 1 (mean 0.6) | 0 or 1 (mean 0.3) |
| Adult male | 1 or 2 (mean 1.2) | 1 or 2 (mean 1.0) |
| Juvenile female | 0 or 1 (mean 0.9) | 0 or 1 (mean 0.4) |
| Yearling female | 0 or 1 (mean 0.7) | 0 or 1 (mean 0.6) |
| Adult female | 2 or 3 (mean 2.1) | 1 or 2 (mean 1.8) |

| **Mortality Rates** Note: these probabilities are adjusted to be linearly inversely proportional to group size so smaller groups have lower mortality rates. The adjustment uses a mortality rate multiplication factor based on the equation: 1 – (0.07 * ([SocialGroupAverage] – [GroupSize])), where SocialGroupAverage is set to 5.7. 6.7. or 7.5 depending on whether the social group has a carrying capacity of 2, 3, or 4 respectively. | |
| --- | --- |
| male 1st 2m pre-emergence | 0.2400 |
| female 1st 2m pre-emergence | 0.2400 |
| male ELISA negative (healthy) | 0.0637 |
| Female ELISA negative (healthy) | 0.0500 |
| Male ELISA positive | 0.0908 |
| Female ELISA positive | 0.0513 |
| Male single site excretor | 0.1168 |
| Female single site excretor | 0.0479 |
| Male multi-site excretor | 0.2831 |
| Female multi-site excretor | 0.1461 |
| **Breeding Probabilities** |  |
| First female | 0.85 |
| 2nd female [adjustable^(1)^] | 0.40 +/- |
| 3rd female [adjustable^(1)^] | 0.40 +/- |
| 4th female [adjustable^(1)^] | 0.40 +/- |
| ^(1)^ Note: the probabilities of 2^nd^/3^rd^/4^th^ female breeding are adjusted to be linearly inversely proportional to group size – so smaller groups may breed back up to size faster. The adjustment is based on the equation: 0.40 + ([GroupSize] - 6.7) * -0.079, but limited between the values 0.00 and 0.85. | |
| **Litter Size Probabilities**  Taken from (p160) |  |
| 1 cub | 0.08 |
| 2 cubs | 0.18 |
| 3 cubs | 0.51 |
| 4 cubs | 0.18 |
| 5 cubs | 0.05 |
| **Dispersal Probabilities** |  |
| Male | 0.009390 |
| Female | 0.000834 |
| **Health-Status Transfer Probabilities**  Taken from |  |
| male ELISA positive to single site excretor | 0.0479 |
| female ELISA positive to single site excretor | 0.0316 |
| male ELISA positive to multi-site excretor | 0.0140 |
| female ELISA positive to multi-site excretor | 0.0094 |
| male single site excretor to multi-site excretor | 0.0727 |
| Female single site excretor to multi-site excretor | 0.0479 |

| **Infection Transmission Probabilities**  Rates set to give a badger prevalence and CHB rate appropriate for each region | **England** | **NI** |
| --- | --- | --- |
| single site excretors |  |  |
| badger to badger within group | 0.036 | 0.0615 |
| badger to badger between group | 0.0018 | 0.003075 |
| badger to cow | 0.000375 | 0.000275 |
| multi-site excretors |  |  |
| badger to badger within group | 0.072 | 1.23 |
| badger to badger between group | 0.0036 | 0.0615 |
| badger to cow | 0.00075 | 0.00055 |
| The rate of transmission between badgers and cattle on neighboring farms is 0.3 times that of the transmission rate between badgers and farms that overlap their own territories. This modification is in force during perturbation. | | |

| **TB-Test probabilities** |  |
| --- | --- |
| TB-Test sensitivity (ELISA positive badger positive) | 34% |
| TB-Test sensitivity (single site excretor badger positive) | 42% |
| TB-Test sensitivity (multi-site excretor badger positive) | 78% |
| TB-Test specificity (healthy badger negative) | 95% |

### Appendix 3.2 Cattle Settings:

| **Stocking Density (Beef) Probabilities**  Calculated from June Census 2004 | **England** | **NI** |
| --- | --- | --- |
| 0.5 cattle per hectare | 0.093 | 0.075 |
| 1.0 | 0.144 | 0.160 |
| 1.5 | 0.190 | 0.185 |
| 2.0 | 0.187 | 0.174 |
| 2.5 | 0.130 | 0.133 |
| 3.0 | 0.089 | 0.090 |
| 3.5 | 0.054 | 0.054 |
| 4.0 | 0.034 | 0.037 |
| 4.5 | 0.022 | 0.037 |
| 5.0 | 0.016 | 0.023 |
| 5.5 | 0.011 | 0.016 |
| 6.0 | 0.007 | 0.010 |
| 6.5 | 0.005 | 0.008 |
| 7.0 | 0.004 | 0.005 |
| 7.5 | 0.004 | 0.005 |
| 8.0 | 0.002 | 0.003 |
| 8.5 | 0.002 | 0.003 |
| 9.0 | 0.002 | 0.003 |
| 9.5 | 0.002 | 0.002 |
| **Stocking Density (Dairy) Probabilities**  Calculated from June Census 2004 |  |  |
| 0.5 cattle per hectare | 0.033 | 0.003 |
| 1.0 | 0.058 | 0.014 |
| 1.5 | 0.115 | 0.045 |
| 2.0 | 0.217 | 0.109 |
| 2.5 | 0.204 | 0.155 |
| 3.0 | 0.151 | 0.193 |
| 3.5 | 0.085 | 0.174 |
| 4.0 | 0.051 | 0.115 |
| 4.5 | 0.028 | 0.064 |
| 5.0 | 0.019 | 0.041 |
| 5.5 | 0.011 | 0.027 |
| 6.0 | 0.010 | 0.013 |
| 6.5 | 0.004 | 0.009 |
| 7.0 | 0.003 | 0.006 |
| 7.5 | 0.001 | 0.005 |
| 8.0 | 0.003 | 0.005 |
| 8.5 | 0.003 | 0.002 |
| 9.0 | 0.000 | 0.003 |
| 9.5 | 0.003 | 0.003 |
| Over-Stock Limit  (the herd-size multiplier which determines the threshold when herd-size must be corrected by "selling-movements"). A value of 1.0 means zero-tolerance to herd-size changes, and cattle are sold/bought the same time-step to correct. | 1.0 | |
| Under-Stock Limit  (the herd-size multiplier which determines the threshold when herd-size must be corrected by "buying-movements". A value of 1.0 means zero-tolerance to herd-size changes, and cattle are sold/bought the same time-step to correct). | 1.0 | |
| **Beef Age/Sex Profile**  Calculated from June Census 2004 |  |  |
| male 1-yr-old | 0.16 | |
| male 2-yr-old | 0.13 | |
| male 3-yr-old | 0.04 | |
| male 4-yr-old | 0.00 | |
| male 5-yr-old | 0.00 | |
| female 1-yr-old | 0.14 | |
| female 2-yr-old | 0.18 | |
| female 3-yr-old | 0.20 | |
| female 4-yr-old | 0.10 | |
| female 5-yr-old | 0.05 | |
| **Dairy Age/Sex Profile**  Calculated from June Census 2004 |  |  |
| male 1-yr-old | 0.07 | |
| male 2-yr-old | 0.05 | |
| male 3-yr-old | 0.01 | |
| male 4-yr-old | 0.00 | |
| male 5-yr-old | 0.00 | |
| female 1-yr-old | 0.13 | |
| female 2-yr-old | 0.22 | |
| female 3-yr-old | 0.30 | |
| female 4-yr-old | 0.15 | |
| female 5-yr-old | 0.07 | |
| Cattle Birth Rate (per 2m time step)  Taken from Defra Stats Report (Economics of Milk Production - England and Wales 2002/03, chapter 5: autumn calvers = 0.93 calves per year, spring calvers = 0.96 calves per year) | 0.159 | |
| **Mortality Rates**  Calculated from CTS slaughter data 2002-2004 | **England** | **NI** |
| beef male, 6months x 1 | 0.0186 | 0.0010 |
| beef male, 6months x 2 | 0.0113 | 0.0290 |
| beef male, 6months x 3 | 0.0821 | 0.2200 |
| beef male, 6months x 4 | 0.0698 | 0.0698 |
| beef male, 6months x 5 | 0.3958 | 0.3958 |
| beef male, 6months x 6 | 0.5479 | 0.5479 |
| beef male, 6months x 7 | 0.1642 | 0.1642 |
| beef male, 6months x 8 | 0.1796 | 0.1796 |
| beef male, 6months x 9 | 0.1573 | 0.3710 |
| beef male, 6months x 10 | 0.2028 | 0.4020 |
| beef male, 6months x 11+ | 0.1565 | 0.0560 |
| beef female, 6months x 1 | 0.0182 | 0.0182 |
| beef female, 6months x 2 | 0.0072 | 0.0072 |
| beef female, 6months x 3 | 0.0145 | 0.0145 |
| beef female, 6months x 4 | 0.1127 | 0.1127 |
| beef female, 6months x 5 | 0.3354 | 0.3354 |
| beef female, 6months x 6 | 0.2353 | 0.2353 |
| beef female, 6months x 7 | 0.1025 | 0.1025 |
| beef female, 6months x 8 | 0.1229 | 0.1229 |
| beef female, 6months x 9 | 0.1238 | 0.1238 |
| beef female, 6months x 10 | 0.1347 | 0.1347 |
| beef female, 6months x 11+ | 0.1807 | 0.1807 |
| dairy male, 6months x 1 | 0.1394 | 0.1394 |
| dairy male, 6months x 2 | 0.0180 | 0.0180 |
| dairy male, 6months x 3 | 0.0974 | 0.0974 |
| dairy male, 6months x 4 | 0.0594 | 0.0594 |
| dairy male, 6months x 5 | 0.3738 | 0.3738 |
| dairy male, 6months x 6 | 0.5367 | 0.5367 |
| dairy male, 6months x 7 | 0.1691 | 0.1691 |
| dairy male, 6months x 8 | 0.1684 | 0.1684 |
| dairy male, 6months x 9 | 0.1258 | 0.1258 |
| dairy male, 6months x 10 | 0.1616 | 0.1616 |
| dairy male, 6months x 11+ | 0.1484 | 0.1484 |
| dairy female, 6months x 1 | 0.0565 | 0.0565 |
| dairy female, 6months x 2 | 0.0116 | 0.0116 |
| dairy female, 6months x 3 | 0.0096 | 0.0096 |
| dairy female, 6months x 4 | 0.0303 | 0.0303 |
| dairy female, 6months x 5 | 0.0500 | 0.0500 |
| dairy female, 6months x 6 | 0.0801 | 0.0801 |
| dairy female, 6months x 7 | 0.0804 | 0.0804 |
| dairy female, 6months x 8 | 0.0910 | 0.0910 |
| dairy female, 6months x 9 | 0.1209 | 0.1209 |
| dairy female, 6months x 10 | 0.1328 | 0.1328 |
| dairy female, 6months x 11+ | 0.1921 | 0.1921 |

| **TB-Test probabilities**   - based on data analysis from Tony Goodchild (UK Veterinary Laboratory Agency) giving sensitivity of 70% for standard interpretation test and 90% for severe interpretation test and ratio of Inconclusive Reactors (IRs) to Conclusive Reactors (CRs) of 1.0 for infected cattle and 0.011 for infectious cattle. - based on data analysis from Tony Goodchild (UK Veterinary Laboratory Agency) giving specificity of 99.7% for IRs (standard and severe test), and 99.935% (standard test) and 99.8 (severe test) for CRs and based on finding that "super-excretor" cattle do not respond to the TB test Tony Goodchild (UK Veterinary Laboratory Agency). | |
| --- | --- |
| **Standard TB-Test probabilities of CR** | |
| health category 1 | 0.0007 |
| health category 2 | 0.4050 |
| health category 3 | 0.8100 |
| health category 4 | 0.0007 |
| health category 5 | 0.4050 |
| health category 6 | 0.4050 |
| **Standard TB-Test probabilities of IR** | |
| health category 1 | 0.003 |
| health category 2 | 0.405 |
| health category 3 | 0.010 |
| health category 4 | 0.003 |
| health category 5 | 0.405 |
| health category 6 | 0.405 |
| **Severe TB-Test probabilities of CR** | |
| health category 1 | 0.002 |
| health category 2 | 0.425 |
| health category 3 | 0.850 |
| health category 4 | 0.002 |
| health category 5 | 0.425 |
| health category 6 | 0.425 |
| **Severe TB-Test probabilities of IR** | |
| health category 1 | 0.003 |
| health category 2 | 0.425 |
| health category 3 | 0.010 |
| health category 4 | 0.003 |
| health category 5 | 0.425 |
| health category 6 | 0.425 |
| TB-Detect Probability at Slaughter (of an infected animal)  Calculated from CTS data | 0.217 |

| **Infection Transmission Probabilities**  Infectious & super-infectious transmission rates not differentiated for cattle. Dairy and Beef are differentiated | |
| --- | --- |
| dairy-cow to cow within-group | 0.007100 |
| dairy-cow to cow between-group | 0.000355 |
| Super-infectious dairy-cow to badger | 0.000050 |
| Beef-cow to cow within-group | 0.014300 |
| Beef-cow to cow between-group | 0.000715 |
| Super-infectious Beef-cow to badger | 0.000050 |
| **Health-Status Transfer Probabilities (Disease progression)** |  |
| male infected to infectious | 0.42 |
| male infected to super- infectious (anergic) | 0.001 |
| female infected to infectious | 0.42 |
| female infected to super-infectious (anergic) | 0.001 |
| male infectious to infected | 0 |
| male infectious to super- infectious (anergic) | 0.001 |
| female infectious to infected | 0 |
| female infectious to super-infectious (anergic) | 0.001 |

**References**

1. Graham J, Smith G, Delahay R, Bailey T, McDonald R, et al. (2013) Multi-state modelling reveals sex-dependent transmission, progression and severity of tuberculosis in wild badgers. Epidemiology and infection 141: 1429-1436.

2. Neal E, Cheeseman C (1996) Badgers. London: T & AD Poyser ltd.

3. Munroe F, Dohoo I (1999) Estimating the incidence rate of within-herd spread of M. bovis. Proceedings of the Society for Veterinary Epidemiology and Preventive Medicine Conference.

4. Fischer E, Van Roermund H, Hemerik L, Van Asseldonk M, De Jong M (2005) Evaluation of surveillance strategies for bovine tuberculosis (Mycobacterium bovis) using an individual based epidemiological model. Preventive veterinary medicine 67: 283-301.
