# Supplementary material for: Model of Selective and Non-Selective Management of Badgers (Meles meles) to Control Bovine Tuberculosis in Badgers and Cattle
Source: PLoS One. 2016 Nov 28;11(11):e0167206. doi: 10.1371/journal.pone.0167206 (PMC5125688; doi:10.1371/journal.pone.0167206)
Supplement: S4 Appendix — (DOC) [file pone.0167206.s004.doc]

**S4 Appendix – Model Processes (submodels)**

1. *Model scheduling*
   Within each two-month time step the following sequential processes occurred:
2. Birth of badgers (time-step 1 only = February)
3. Ageing of badgers (time-step 1 only = February)
4. Birth of cattle
5. Mortality of badgers
6. Mortality of cattle (sent to slaughter)
7. Dispersal of badgers
8. Control of badgers (default: years 120 to 124 inclusive, and time-step 3 only = June)
9. Perturbation of badgers
10. Movement of cattle (farm to farm) including pre-movement testing of cattle, if included
11. Spread of bTB infection – first badger-to-badger, then cattle-to-cattle, badger-to-cattle, and cattle-to-badger
12. bTB disease progression in badgers
13. bTB disease progression in cattle
14. Cattle testing (routine testing of cattle for bTB, if due)
15. Ageing of cattle
16. Output data (time-step 6 only – end of each year)

The above processes (sub-models) are described in more detail elsewhere , along with a description of the processes allowing population and disease dynamics to stabilize before badger control is simulated.

1. *Creation of badger territories*
   A specified number of badger territories are created: main setts are placed randomly across the grid, and grid squares allocated to closest main sett to give fully contiguous tessellated badger groups. The grid is treated as a torus so there are no edges. The total number of badger groups added to the grid (see Appendix S2) gives an average territory size of 1.33km^2^ (England) and 1.78 km^2^ (Northern Ireland) giving territory densities of 0.75km^-2^ and 0.56km^-2^.
2. *Creation of Farms*A specified number of farms are added to the grid to produce a realistic farm density of 0.78km^-2^ (England) and 1.75km^-2^ (Northern Ireland). Farm “centroids” are added to the grid at random, and allocated as a farm type (beef, dairy, mixed, X1, X2, X3, X4) stochastically to give the correct proportions of farm-types. X1-X4 represent farms that also have other stock and hence are allocated as beef or dairy, but with proportionately smaller grazing areas for cattle. Potential farmland for grazing allocation is determined by tessellation from the centroids.
3. *Creation of grazing areas*The grazing area for each farm is determined according to preset proportions of grazing land for each farm-type (see Appendix S2). Sufficient grid squares are marked as grazing land by using a spiraling algorithm starting from a random grid square within the farmland. Thus, all grazing area is contiguous within a farm, but not necessarily between farms.
4. *Definition of “near-farms”*The distance from each farm’s grazing area centroid to every other is calculated, sorted, and stored in a matrix for later use in the “Move Cattle” procedure, so that farms buy cattle from nearby farms in preference to distant farms.
5. *Creation of parishes*A specified number of parishes are created (see Appendix S2) by randomly allocating grid-squares as parish centroids, then tessellating around each centroid. The mean simulated parish size is 13.3km^2^ in both England and Northern Ireland.
6. *Definition of neighbors*This procedure determines the badger neighbors of each badger territory, the cattle herd neighbors of cattle herds (where neighbouring grazing areas are contiguous), and which cattle herds overlap which badger territories. This allows between-group TB transmission to be simulated. Badger-to-cattle and cattle-to-badger transmission only occurs where the grazing and the badger territory overlap (as opposed to being simply adjacent).
7. *Definition of neighbours during perturbation*

The definition of the neighborhood relationship between badgers and cattle changes when culling related perturbation is in effect and badgers may come into contact with farms that overlap with neighboring badger territories as well as those that overlap their own territory. This procedure determines the additional farms that neighbor each badger group when they are experiencing perturbation and the badger territories that overlap each farm when perturbation is in effect. The transmission rate from badgers to cattle in these more distant farms is adjusted to be 0.3 times that to directly overlapping farms.

1. *Addition of badgers*Badgers are added to each badger territory at the start of year one, with some stochastic options (see Appendix S1), to give a stable mean badger group size of about 6.7 (England) and 4.4 (Northern Ireland) adult badgers per group, as measured at the end of December.
2. *Addition of cattle*Individual herd size is calculated from the grazing area on each farm, and a stocking density taken at random from the stocking-rate distribution (see Appendix S2). Cattle are then added stochastically, using probabilities based on the profile of ages/sexes for the herd type. For simplicity, all cattle are initially allocated to ages equivalent to the first time-step of each year. Cattle are added to each farm at the start of year 1, but kept static till year 20. The simulated stable mean herd sizes are about 44 (England) and 74 (Northern Ireland) head for beef, and about 86 (England) and 185 (Northern Ireland) head for dairy.
3. *Births of badgers*In the model female badgers give birth in the first time step of each year, which is equivalent to January + February. The number of females that breed in any one badger group is determined probabilistically (see Appendix S3), although this is limited by the number of 2+ yr-old females, and the carrying capacity of the group. The breeding probability for the first female is fixed, but the probabilities of the 2nd/3rd/4th are higher for groups with fewer badgers present (linear relationship). Litter sizes are also determined probabilistically (see Appendix C.1), mean litter size is 2.94, and the cub male : female ratio is 1:1.
4. *Ageing of badgers*
   This occurs in the first time step of each year within the “birth of badgers” procedure. All badgers are aged by one year immediately after the birth routine, but just before the new cubs are added to the main population array.
5. *Mortality of badgers*Badger mortality rates , are dependent on sex, age, and health status (see Appendix S3), and are adjusted linearly to give lower mortality rates for smaller groups. The mortality rates are applied to individual badgers probabilistically.
6. *Dispersal of badgers*Dispersal probabilities are sex-dependent (see Appendix S3), but are not related to age or season. The dispersal routine occurs every time-step. Badgers disperse only as far as their neighboring group, and tend to move to a group with fewer badgers if one is available. Badgers are not allowed to disperse twice in one time-step.
7. *Social perturbation of badgers*This procedure moves badgers to fill vacancies. It occurs whether or not badger control is being simulated, but obviously these perturbation movements are more frequent immediately following badger removal. Sexes are checked independently, groups that already have two of a sex would not receive a third, and the donor group must also have at least three more badgers of that sex than the recipient group. Badgers are moved shorter distances in preference, and a badger is not allowed to make two moves within the same time-step. Note: for details of the simulated “perturbation effect”, see “Transmission of TB – badger to badger” below.
8. *Seeding of TB in badgers*At the start of year 20, each badger group is given a high probability to have one badger of random sex and age to be infected with TB. Each selected badger during this seeding process is given a TB status of “infected”.
9. *Seeding of TB in cattle*At the start of year 20 about 10% of farms are chosen at random, and in each of these a single cow of random sex and age is transferred from healthy to infected disease status.
10. *Set timing for annual cattle-test*At the start of year 20, each herd is allocated a random time-step (between 1 and 6) to determine when it will be due for its annual TB-test.
11. *Birth of cattle*
    All female cattle aged over 22 months give birth to one calf annually, on their birthday. The sex ratio of the calves is set at 1:1, the sex being determined probabilistically. Over the age of 60 months, since all cattle still alive remain in that age category, births are determined probabilistically each time-step, using a 2-monthly birth rate. Births are applied to Main herds and Isolated herds in the same way.
12. *Mortality of cattle*Mortality only applies to cattle going to slaughter. For simplicity, natural mortality on the farm is not modeled. Each cow is categorized by age into 6-month periods, and mortality rates applied probabilistically. The mortality rates are dependent on herd-type, sex, and age, but independent of health status (see Appendix S3). Mortality rates were calculated from the CTS data through construction of life-tables (Appendix S3). If a cow going to routine slaughter is infectious or super-infectious (not just infected) a probability of TB detection at slaughter is applied (see Appendix S3), and if TB is detected, movement restriction and testing is triggered at the farm of origin. Mortality is applied to Main herds and Isolated herds in the same way.
13. *Movement of Cattle*If herd-size (sum of main + any isolated herd) is smaller or larger than the ideal size (according to stocking density) extra cattle are moved on or off the farm. All spare cattle for all farms are initially moved into a holding stock (market equivalent), with males moving off a dairy farm as priority, and females off a beef farm, but otherwise picked at random (i.e. independent of age and health-status). Extra animals are then chosen at random to send to “market” to ensure that 40% of cattle move each year. Cattle are then moved from “market” to farms that are short of cattle, females moving to dairy, and males to beef as first priority, then proximity to donor farm as the second priority. Following all within-grid movements, if more cattle are needed, they are added into the grid, and if there are cattle left in the “market”, they are removed to simulate movement from T1 to T3 and T4 areas. Each cow being moved into the grid is given a probability of being infectious with TB, calculated from the proportion of Britain that is T1, and the cattle TB prevalence in the model at the time of the move. The number of infected cattle being moved outside the grid is used to calculate the extra CHBs that could be caused by those movements (e.g. from T1 to T4 areas). After year 99, if pre-movement testing (PrMT) is in place, all cattle in T1 and T2 areas are tested before movement (see “Test Cattle pre-movement”, below), and if any test from a farm is positive, movement is not allowed and standard testing procedures are triggered.
14. *Transmission of TB – badger to badger*Each infectious badger has a chance of infecting every contact, both within-group, and between-group (neighbors). Transmission rates are set higher for super-infectious badgers, and between-group rates are set to 5% of within group rates (see Appendix S3 and ). During years of badger control, between-group infection rates are recalculated (see “*Setting Transmission Rates*” below) to give higher TB transmission rates in/around the control area (i.e. a higher probability of between-group contacts). This is to simulate the “perturbation effect” of badger culling.
15. *Transmission of TB – cattle to cattle*Each infectious cow has a chance of infecting every contact, both within-group, and between-group (neighbors). Probabilities of transmission are currently the same for infectious and super-infectious; and between herd rates are set to 5% of within herd rates (see Appendix S3). Transmission rates from beef cattle are set to about twice the value of dairy cattle . Unconfirmed reactors that are separated from the main herd (put together with other unconfirmed reactors in an isolated field on the farm) are classified as “isolated herds” in the model (herds isolated as part of the TB control procedures). Such isolated cattle in the model are able to transmit TB infection to each other within the isolated herd, but do not transmit TB infection to any of the healthy cattle in the farm’s main (non-isolated) herd, or to any cattle on neighboring farms. However, it is assumed that badgers still have access to the field holding the isolated cattle, so TB transmission is still able to occur between cattle and badgers.
16. *Transmission of TB – badger to cattle*Each infectious badger has a chance of infecting every contact cow that grazes on land shared by the badger (i.e. where the badger territory and the grazing land overlap – but note that this definition of overlap changes during control-related perturbation). Multi-site excretor badgers are given a transmission rate double that of single-site excretor badgers. (see Appendix S3).
17. *Transmission of TB – cattle to badger*Each infectious cow has a chance of infecting every contact badger where the badger territory overlaps the grazing land. Only super-infectious cows are given a transmission rate greater than zero, and it is set to the same transmission rate as from badger to cow (see Appendix S3).
18. *Setting transmission rates (including perturbation effect)*Transmission rates are applied stochastically, and default values are listed in Appendix S3. Special rates, however, are applied to those badger groups subjected to culling, and to their immediate neighbors. This is to simulate higher contact rates during a period of social perturbation as a result of the culling. This perturbation effect lasts one year following culling so is in effect from the first cull to one year after the last cull in the five year culling period. is simulated during the whole period of culling (five years), and for three years after the last cull (i.e. a total of eight years). Wherever and whenever the perturbation effect is applied, all badger-to-badger between-group transmission rates are increased to equal the within-group rates. Badger-to-cattle, cattle-to-badger, and cattle-to-cattle rates are not adjusted.
19. *Disease progression in badgers*Badgers with TB are given the chance of transferring from one TB-status to another, according to pre-set probabilities (see Appendix S3). A badger can only make one such change per time-step. Disease progression is from ELISA positive to single site excretor to multi-site excretor and all changes are unidirectional. A newly ELISA positive badger does not itself have the chance to infect another badger or cow until the following time-step.
20. *Disease progression in cattle*Cattle with TB are given the chance of transferring from one TB-status to another, according to pre-set probabilities (see Appendix S3). Disease progression is from infected to infectious to anergic. Infected cows also have a possibility of transferring straight to the anergic state in one time-step. Infectious and super-infectious cattle are not able to revert to a lower disease state, and if a cow becomes anergic, it stays in that state till death. A newly infectious cow does not itself have the chance to infect another cow or badger until the following time-step.
21. *Testing of Cattle*This procedure simulates both routine testing and TB-triggered testing. A countdown system is used to trigger the routine “whole-herd” tests for each farm at the appropriate time-step, and a different countdown for “partial-herd” tests if there are isolated cattle. For the whole-herd test both Main and Isolated cattle are tested. Every cow is tested using probabilities to determine whether it will be a Reactor or Inconclusive (see Appendix S3). These test probabilities are dependent on cow health-status and test-type (standard or severe interpretation). Inconclusive (unconfirmed) reactors are modeled to simulate the processes that would occur in the field (isolation, movement restrictions, test follow-ups) including economic costs. Inconclusives are isolated, but any individuals testing Inconclusive for the third time running are classed as Reactors. Any Reactors are slaughtered and subject to *post mortem* examination. It is assumed that all infected reactor cows will be confirmed at *post mortem*, flagging up a confirmed CHB and triggering movement restrictions on contiguous herds, and their testing in the next time-step. Test results are analyzed on a herd-basis, and a herd’s test-status and next test requirement stored. When a test is positive a series of procedures are brought into effect, simulating the veterinary procedures that are used in the field. If tests are negative, and appropriate, isolated cattle rejoin the main herd.
22. *Test cattle – pre-movement*After pre-movement testing has been switched on, this procedure is called whenever cattle are about to be moved, and all cattle over a specified age are tested. Only the cattle about to be moved are tested, and in the first year, of pre-movement testing only cattle of age 16 months or older, and from the second year onwards only cattle aged 2 months or older. The actual values used in the field are 15 months for the first year and 6 weeks from the second year onwards, but the values of 2 months and 16 months are used in the model to fit in with the model’s 2-month time-step. If any animals react positive to the pre-movement TB test, all animals of that herd are stopped from moving, and a series of procedures are brought into effect, simulating the veterinary procedures that are used in the field. If all tests are negative then the cattle are allowed to move (to market). Pre-movement testing is not applied to cattle moving straight to slaughter. Note that pre-movement testing is only applied in England, not Northern Ireland.
23. *Ageing of cattle*Every two-month time step all cattle are aged by two months, except those already aged to the maximum category of 30 (60 months = 5 yrs), which simply stay in that category till death.
24. *Switch Test Intervals*This procedure allows farms in a parish to switch their test-interval status according to their CHB rate history. It is applied at the end of each year, from year 50 onwards, and the average number of herds that have had a breakdown within the previous six years is calculated for each parish. This determines what Test Interval all the farms in a parish should be (T1 to T4) (see Appendix S1 for details of the algorithm). In the model, a parish can only change by one category in any one year (e.g. T1 to T2, but not T1 to T3). A new test month is calculated for each farm at the end of each year, dependent on any test interval change and when the next test was due, and this new test month applies immediately.
25. *Mark Farms for Management (include setting staging reducing and compliance)* The worst parish in terms of CHB rates is selected first, by calculating the CHB density from years 117 to 119. Then neighbouring farms are selected until the management area has “grown” to the required size. If the compliance of land access is to be less than 100%, the required number of selected farms are then removed at random to simulate the non-compliant farms.
26. *Mark badger groups for management (include drawing-out calculations and reducing permeability)*

The badger groups to be treated are determined by first selecting the farms to apply management, according to the spatial strategy (see “Switch Test Intervals” above). Then the proportion of each badger group that overlapped farmland selected for treatment is calculated, and the trapping efficacy calculated from “drawing-out” rules to permit a proportion of badgers to be caught when only a proportion of the territory is available for trapping.

1. *Apply badger control*Each control method is applied for five years from year 120 to year 124 inclusive, starting with identical conditions to the no-control option within that simulation at the start of year 120, to give a fair comparison between the control methods. The badger groups to be controlled are determined by selecting the parish with the highest CHB density (from years 117 to 119) and selecting the appropriate number of parishes located contiguously around it. If there are choices, the parishes with the highest CHB densities are selected. A proportion of farms are excluded at random from having badger control, to simulate non-compliance. Control of the selected badger groups is applied stochastically at the specified control rate for the method (see Section 6.1.1 for control rates simulated) every third time-step (equivalent of May/June) once per year for the five years.
2. *Save data*
   Output parameters are calculated at the end of each year, including badger population, badger TB (number and prevalence), cattle herd breakdown rates. All output data for each set of simulations is saved in one Excel file.

**References**

1. Wilkinson D, Bennett R, McFarlane I, Rushton S, Shirley M, et al. (2009) Cost-benefit analysis model of badger (Meles meles) culling to reduce cattle herd tuberculosis breakdowns in Britain, with particular reference to badger perturbation. Journal of Wildlife Diseases 45: 1062-1088.

2. Graham J, Smith G, Delahay R, Bailey T, McDonald R, et al. (2013) Multi-state modelling reveals sex-dependent transmission, progression and severity of tuberculosis in wild badgers. Epidemiology and infection 141: 1429-1436.

3. Smith GC, Cheeseman CL, Wilkinson D, Clifton-Hadley RS (2001) A model of bovine tuberculosis in the badger *Meles meles*: the inclusion of cattle and the use of a live test. Journal of Applied Ecology 38: 520-535.

4. Munroe F, Dohoo I (1999) Estimating the incidence rate of within-herd spread of M. bovis. Proceedings of the Society for Veterinary Epidemiology and Preventive Medicine Conference.
